# Semi-Supervised Seven-Segment LED Display Recognition with an Integrated Data-Acquisition Framework

**DOI:** 10.3390/s26010265

**Published:** 2026-01-01

**Authors:** Xikai Xiang, Chonghua Zhu, Ziyi Ou, Qixuan Zhang, Shihuai Zheng, Zhen Chen

**Affiliations:** 1 College of Mechanical and Energy Engineering, Guangdong Ocean University, Yangjiang 529500, China; 2 College of Engineering and Information Engineering, Guangdong Ocean University, Zhanjiang 524088, China; 3 State Key Laboratory of Mechanical Transmission for Advanced Equipment, Chongqing University, Chongqing 400044, China

**Keywords:** LED segment displays, convolutional neural network, semi-supervised learning, adversarial training, Squeeze-and-Excitation B Block

## Abstract

In industrial inspection and experimental data-acquisition scenarios, the accuracy and efficiency of digital tubes, which are commonly used display components, directly affect the intelligence of the system. However, models trained on data from specific environments may experience a significant drop in recognition accuracy when applied to different environments derived from impacts of various specific scenarios (e.g., temperature changes, changes in light intensity, changes in rate, and color contrast between equipment displays and environments, among others), which may affect model accuracy. To ensure recognition accuracy, we may need to collect data from specific environments to retrain the model for each specific environment, but manual annotation is often inefficient. To address these issues, this article proposes a solution integrating image processing with deep learning within specific scenarios, encompassing the entire workflow from data acquisition to model training. Employing image processing techniques to provide high-quality training data for models, we construct a semi-supervised adversarial learning framework based on an improved self-training algorithm. The framework employs the k-means clustering algorithm for stratified sampling preparation, adds the Squeeze-and-Excitation B Block to the Convolutional Neural Network backbone, and employs the Adversarial Generative Adversarial Network to generate adversarial examples for adversarial training, thus enhancing both classification accuracy and robustness.

## 1. Introduction

In industrial automation and intelligent monitoring systems, the automatic and accurate reading of digital information from display devices is of great significance to the realization of data intelligence. However, in real-world scenarios, image recognition is often affected by various disturbances such as varying illumination, motion blur, and complex background noise [1,2,3,4]. Although deep learning models have achieved remarkable success in image recognition, their performance is highly dependent on large-scale, high-quality annotated datasets [5,6]. In industrial practice, acquiring sufficient and accurately labeled data for every possible working and environmental condition is often demanding and impractical [7]. Moreover, deep Convolutional Neural Network (CNN) image recognition models are easily deceived by tiny adversarial perturbations that are visually imperceptible [8,9]. Since training a general model to adapt to all environments is impractical, we turn to training specialized models for specific environments. To overcome the heavy workload of repetitive data collection and labeling in this strategy, this paper proposes a method that automatically detects LED segment display data to quickly expand the dataset in a specific environment and build semi-supervised LED segment display recognition models for specific scenarios, thereby reducing the workload of manual labeling. To address the core barrier of inefficient manual data annotation, semi-supervised learning has shown considerable potential by collecting abundant unlabeled data to improve model generalization [10]. Among semi-supervised learning, pseudo-labeling is a simple and effective approach that assigns high-confidence model predictions on unlabeled data as training labels, thereby expanding the training set [11,12]. However, a major limitation of this technique is the confirmation bias, whereby incorrect predictions made in the initial phase are reinforced during subsequent training, thereby constraining overall model performance. To address this problem, researchers have introduced clustering-based strategies to reduce the impact of such biased predictions [13] and uncertainty-aware mechanisms [14] to select more reliable pseudo-labels and reduce the accumulation of noisy labels. Even in a fixed scene, micro-level physical disturbances accumulate over time and create distribution drift. Industrial applications demand long-term stability and minimal maintenance cost; therefore, robustness is essential. Adversarial training simulates the worst perturbations, allowing the model to remain stable under realistic real-world variations while still being optimized for a specific environment. In other research domains, adversarial training and related generative techniques have been demonstrated to significantly improve model robustness [15]. By learning the adversarial samples after iterative training, models are encouraged to develop more stable and discriminative feature representations [16]. Generative adversarial networks and their variants can synthesize samples that closely resemble the real data distribution [17,18]. This not only enriches data augmentation methods for semi-supervised training but also provides the simulation of complex noise conditions in low-data environments [19,20].

Text detection methods in natural images can be divided into two categories: region-based methods [21,22,23] and texture-based methods [22]. The former utilizes similar character components, such as color [21,22], stroke width [23], and edges [24]. Based on these similarities, pixels are connected to form a region. Subsequently, non-text connected components are filtered out by a classifier. This paper uses the region-based method as the foundation for the automatic detection approach. In the last decade, with the advancement of deep neural networks (especially CNNs), the overall performance of automated fine-grained image classifiers has improved significantly. For readers unfamiliar with the principles of deep learning and CNNs, we recommend referring to the work of Goodfellow et al. [25]. The success of deep learning models trained under fully supervised conditions typically relies on the availability of large-scale annotated image databases. For LED segment display recognition, such extensive datasets already exist, thanks to citizen science and open data initiatives [26]. To validate the applicability to datasets from diverse environments, we have also collected a large-scale dataset for experimental purposes.

Integrating the strengths of semi-supervised learning and adversarial training to build a robust recognition framework for addressing annotation scarcity and environmental disturbances represents a highly valuable research direction. Drawing on ideas validated in domains such as fault diagnosis [27,28] and medical image analysis, we seek to achieve robust recognition performance under minimal annotation budgets. Since many legacy assets, such as the JK-50A power supply in this study, lack digital communication ports, vision-based recognition provides a non-intrusive “optical data diode”. This approach maintains a physical air gap between the equipment and the network, ensuring that data can be collected without creating a pathway for cyber threats. This study explores a semi-supervised framework that integrates optimized pseudo-label selection, clustering, and adversarial data augmentation into a streamlined workflow from acquisition to training. Our results demonstrate that this improved algorithm achieves superior recognition rates and efficiency over traditional methods, handling multi-source digits with high generalization and robustness. By improving the accuracy of label correction and reducing manual labeling time by over 90%, this approach is particularly advantageous for large-scale industrial dataset labeling. The main contributions of this work are threefold: (1) It simplifies the workflow from dataset acquisition to model training in specific industrial scenarios; (2) It enhances the accuracy of pseudo-label correction through a K-means stratified sampling strategy; (3) It improves model robustness against environmental disturbances using AdvGAN++. The remainder of this paper is organized as follows: Section 2 describes image preprocessing and the proposed framework; Section 3 presents the experimental results and ablation studies; and Section 4 concludes the study and discusses future research directions.

## 2. Materials and Methods

This section details the proposed integrated framework, covering the industrial rationale for non-invasive recognition, image preprocessing techniques, and the core semi-supervised adversarial architecture. The methodology is designed to balance high recognition accuracy with minimal labeling overhead in complex industrial environments.

### 2.1. Image Preprocessing

During image capture, noise points may appear, adversely affecting the performance of edge detection algorithms. To mitigate this, a Gaussian filter smooths the image, reducing noise points and enhancing edge detection accuracy. After filtering, the image exhibits a softer overall visual appearance and gentler color transitions. This preprocessing step minimizes interference in subsequent feature extraction. The one-dimensional zero-mean Gaussian function is represented as follows:

(1) $$\begin{array}{c} g(x)=e^{\frac{-x^{2}}{2\sigma^{2}}}, \end{array}\;$$

where the Gaussian distribution parameter σ determines the width of the Gaussian filter.

In image processing, the two-dimensional zero-mean discrete Gaussian function is commonly used as a smoothing filter:

(2) $$\begin{array}{c} G(x,y)=Ae^{\frac{{-(x}^{2}+y^{2})}{2\sigma^{2}}}=Ae^{\frac{-r^{2}}{2\sigma^{2}}}. \end{array}\;$$

Sampling and quantizing the above continuous Gaussian distribution, followed by normalization of the template, obtains the discretization stencil:

(3) $$\begin{array}{c} G^{3}=\frac{1}{16}\left(\begin{array}{ccc} 1 & 2 & 1\\ 2 & 4 & 2\\ 1 & 2 & 1 \end{array}\right) \end{array}.$$

Subsequently, extract edge features accurately from the processed image and employ the Sobel operator to compute the first-order derivatives in the horizontal and vertical directions, denoted $G_{x}$ and $G_{y}$, respectively. From these gradient components, the gradient magnitude and direction of the boundaries can be further derived. The specific formulas are as follows:

(4) $$\mathrm{Edge}(G)=\sqrt{G_{x}^{2}+G_{y}^{2}},$$

(5) $$\mathrm{Angle}(\theta )=\arctan\left(\frac{G_{y}}{G_{x}}\right),$$

(6) $${\;K}_{x}=\left[\begin{array}{ccc} -1 & 0 & 1\\ -2 & 0 & 2\\ -1 & 0 & 1 \end{array}\right],$$

(7) $${\;K}_{y}=\left[\begin{array}{ccc} -1 & -2 & -1\\ 0 & 0 & 0\\ 1 & 2 & 1 \end{array}\right],$$

where $K_{x}$ denotes the horizontal convolution kernel, $K_{y}$ denotes the vertical convolution kernel, $G_{x}$ denotes the horizontal gradient, $G_{y}$ denotes the vertical gradient.

Applying the Sobel operator to the processed image, the first-order derivatives in the horizontal and vertical directions ($G_{x}$ and $G_{y}$) are computed. Next, by applying gradient maps to determine the gradient magnitude and direction of the boundary, we remove non-edge points, resulting in thinner boundaries. To identify the real boundaries, two thresholds are defined, namely minval and maxval. Pixels with gray gradients exceeding maxval are classified as boundaries, while any below minval are discarded, and those with values in between are connected to the real boundary and considered boundary points.

Applying a mask processes the image, extracting the red regions and calculating the minimum bounding rectangle of all red regions. Within this bounding rectangle, a black border is detected, and Canny border detection is used to obtain more detailed boundary information for further image analysis or feature extraction. Subsequently, an appropriate black border is cropped by setting a specific ratio range, ensuring that the red mask area is fully included. Then, the cropped image is processed with non-maximum suppression again.

### 2.2. Screening Model and Data Generalization

To efficiently extract the cropped data, we employ a combination of red channel enhancement and Convolutional Neural Network (CNN)-based feature learning. Digital tubes, as typically red-light-emitting structures, are distinguished by this feature. Utilizing this significant red feature, the preprocessing step suppresses the brightness of the green and blue channels in the image, thereby enhancing the prominence of red regions. In this process, the image features of the red areas are enhanced while effectively suppressing the mixed color interference of digital tubes. This study applies a binary classification screening model based on CNN. The output layers utilize the sigmoid, formulated as follows:

(8) $$\mathrm{sigmoid}(x)=\frac{1}{1+e^{-x}}.$$

To enhance sample diversity, images are captured and processed using affine transformations under various angles and lighting conditions. Specifically, to reflect the measured physical illuminance range of 300 to 3000 Ix, the contrast gain and brightness are adjusted between 0.8 and 1.2. For geometric variations, the rotation angle is set to ±15°, while the translation operations—which are the primary focus of this study—are limited to 10% of the image dimensions to mimic potential sensor displacement. Incorporating these extensive samples significantly improves the robustness of the cluster. The transformation matrix M is formulated as follows:

(9) $$M=[\begin{array}{ccc} 1 & 0 & c_{x}\\ 0 & 1 & c_{y}\\ 0 & 0 & 1 \end{array}][\begin{array}{ccc} \cos\theta & -\sin\theta & 0\\ \sin\theta & \cos\theta & 0\\ 0 & 0 & 1 \end{array}][\begin{array}{ccc} 1 & 0 & -c_{x}\\ 0 & 1 & -c_{y}\\ 0 & 0 & 1 \end{array}].$$

**Figure 1:**
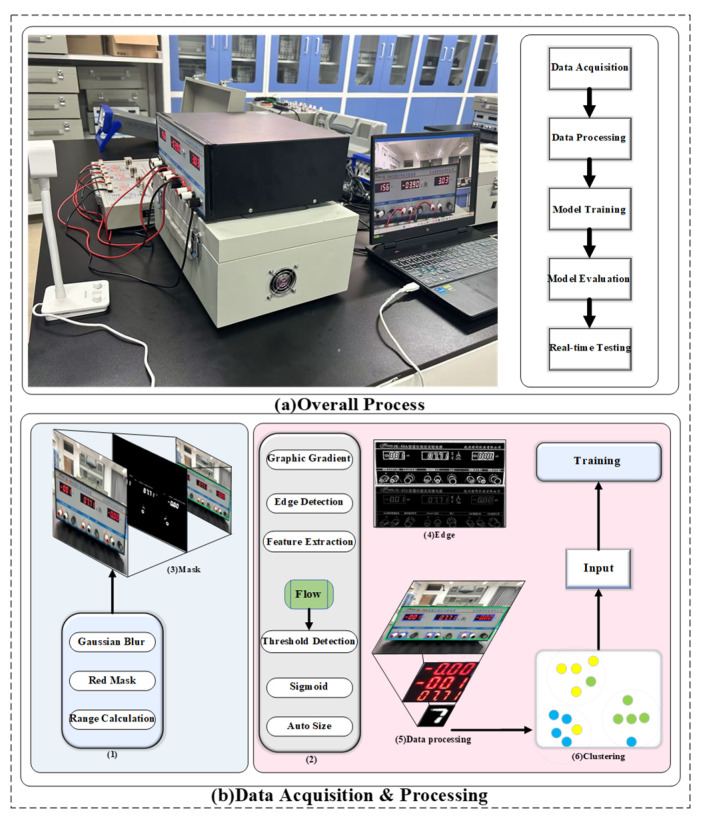
Image Data flowchart.

The general flowchart is depicted in Figure 1:

### 2.3. The Framework of the Semi-Supervised Model

Self-training is a classic semi-supervised learning approach that utilizes a small set of annotated data and a large amount of unlabeled data to improve model performance. In this method, an initial model is used to predict the unlabeled data, and high-confidence predictions are selected as pseudo-labels and put into the training set for retraining the model, then employing the k-means clustering algorithm (K-means) to uniformly sample from each class.

In this study, we adopted an improved self-training framework. Within the adversarial self-training framework, the classifier, clustering algorithm, and adversarial generator share a CNN+SE feature extractor. The training process consists of initial supervised preparation and cyclic self-training. During the supervised preparation phase, the small set of annotated samples is used to train the classifier while updating the backbone and SE module at the same time to obtain an initial feature representation. Each self-training cycle successively involves pseudo-label generation, classifier update, re-clustering, Adversarial Generative Adversarial Network adversarial attacks algorithm training, and optional adversarial-enhanced fine-tuning. This approach generally ensures synergistic evolution between the generator and the classifier, as well as coordinated interaction between clustering and representation learning. The SE module is updated exclusively during classifier training to maintain stability and robustness.

**Figure 2:**
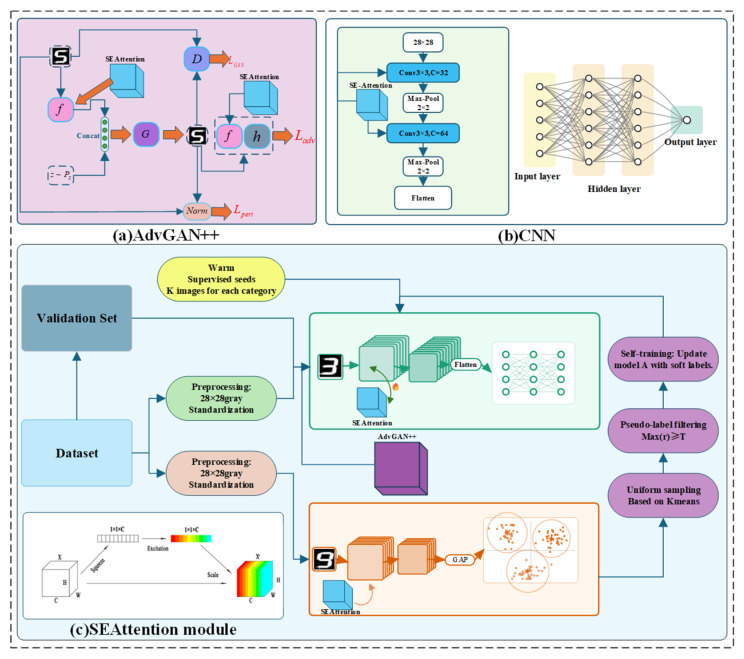
Flowchart of the multi-model fusion.

This study employs the semi-supervised model integrating CNN-Squeeze-and-Excitation B Block (SE) and clustering, and the process and calculation procedure are illustrated in Figure 2:

### 2.4. CNN-SE Model

CNNs have achieved remarkable success as deep learning models in the field of computer vision. Their design is inspired by biological visual systems, aiming to simulate human visual processing. Furthermore, CNNs integrated with SE-Attention have demonstrated significant progress across image recognition, object detection, image generation, and other domains. It becomes an important part of computer vision and deep learning research.

We adopt ReLU as the activation function, which has the capability to perform different linear operations in different regions, such that the overall input-output relationship is no longer a single straight line but rather a polyline with corners. When numerous and multi-layered ReLU neurons are combined, each layer adds new corners and regional divisions based on the preceding output layer of the preceding layer. Through this process, countless simple polyline segments can be pieced together to form arbitrarily complex curves, thereby enabling the network to achieve nonlinear fitting and for output layers to utilize the SoftMax function. The functions are defined as follows:

(10) $$f(x)\;=\max(0,x)\;=\{\begin{array}{cc} 0 & \mathrm{if}\;x\;<\;0\\ x & \mathrm{if}\;x\;\geq \;0 \end{array},$$

SoftMax:

(11) $$y_{k}\;=\;\mathrm{SoftMax}(z{)}_{k}=\frac{e^{z_{k}-z_{\max}}}{\sum_{i=1}^{C}e^{z_{i}-z_{\max}}}\;(k\;=\;1,2,\ldots ,C).\;$$

The squeeze, excitation, and scale operations of the SE block are defined as follows [29]:

(12) $$z_{c}=F_{\mathrm{sq}}(u_{c})=\frac{1}{H\times W}\sum_{i=1}^{H}\sum_{j=1}^{W}u_{c}(i,\;j),$$

Excitation [29]:

(13) $$\begin{array}{c} s=F_{\mathrm{ex}}(z,\;W)=\sigma (g(z,\;W))=\sigma (W_{2}\delta (W_{1\;}z)) \end{array},\;$$

Scale [29]:

(14) $$\begin{array}{c} {\tilde{u}}_{c}=F_{\mathrm{scale}}(u_{c},s_{c})=s_{c}\cdot u_{c} \end{array}\;,\;$$

Squeeze-and-Excitation Block [29]:

(15) $$\mathrm{SE}(X)=F_{\mathrm{scale}}(X,\;\sigma (W_{2}\mathrm{ReLU}(W_{1}F_{\mathrm{sq}}(X)))).\;$$

### 2.5. Clustering Model

**Figure 3:**
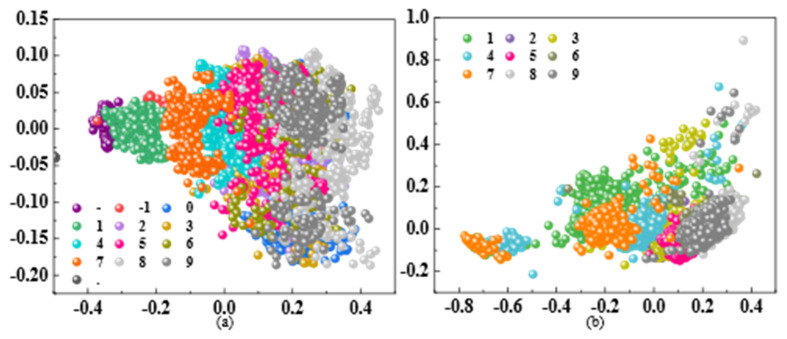
Clustering effect. (**a**) Diagram of Left: Display clustering of the local dataset. (**b**) Diagram of Right: Display clustering of the public dataset.

In this study, the feature extractor employs a hierarchical feature extractor based on CNN and integrates with the SE-Attention mechanism. The input 28 × 28 pixel image undergoes two consecutive rounds of convolution and pooling operations, mapping the original pixel space into a 64-dimensional discriminative feature vector to provide a representation for subsequent clustering analysis. Employing PCA dimensionality reduction techniques, when the proportion of variance explained by the principal components remains above 95%, perform cluster analysis within this space [30], and the clustering is shown in Figure 3.

### 2.6. Adversarial Training Module

CNNs possess formidable fitting capabilities, enabling the formation of rich data representations. However, this very characteristic also carries inherent risks. For instance, applying a minute perturbation δ to the original input x may cause significant alterations in the model’s feature representations, thereby triggering classification or detection errors.

To enhance a model’s resilience against such adversarial attacks, researchers have proposed adversarial training methods [31,32,33,34]. The core strategy involves training models using robust adversarial examples, thereby endowing the trained model with resistance to attacks. Within adversarial generative adversarial networks, the generator maps clean samples to adversarial perturbations, which are subsequently added to the corresponding clean samples. The discriminator’s task is to determine whether an input sample constitutes an adversarial example. Adversarial example generation is as follows:

(16) $$x_{adv,i}={\mathrm{clip}}_{\left[a,b\right]}(x_{i}+\varepsilon \tan h(G(f\left(x_{i}\right),z_{i}))),\;$$

where $x_{i}$ is the normalized input image in the mini-batch, $z_{i}∼N\left(0,I\right)$ is its noise, $f\left(x_{i}\right)$ is the spatial mean of the classifier’s backbone feature map, G is the generator, *ε* caps the perturbation size, ${\mathrm{clip}}_{\left[a,b\right]}(\cdot )$ clamps pixels to [a,b], and $x_{\mathrm{adv},i}$ is the resulting adversarial image, discriminator loss [15]:

(17) $${\;L}_{D}=E[\max(0,1-D(f(x_{i})))]+E\left[\max\left(0,1+D\left(f\left(x_{\mathrm{adv},i}\right)\right)\right)\right],\;$$

where D is a discriminator operating on feature vectors f(·), E[·] denotes the empirical expectation over samples i (and noises $z_{i}$), and the hinge terms encourage $D\left(f\left(x_{i}\right)\right)\geq 1$ for real features and $D\left(f\left(x_{\mathrm{adv}\;,i}\right)\right)\leq -1$ for adversarial features, *KL* combats loss [15]:

(18) $${\;L}_{\mathrm{adv}}=-\mathrm{KL}\left(p(x_{i})\|q\left(x_{\mathrm{adv},i}\right)\right),\;$$

where $\gamma ,\;\lambda_{\mathrm{TV}},\;\lambda_{1}\;\geq \;0$ are scalar weights, $L_{\mathrm{GAN}}=-E\left[D\left(f\left(x_{\mathrm{adv},i}\right)\right)\right]$ is the generator’s hinge-GAN term, $L_{TV}$ is the total-variation penalty on the perturbation $\delta_{i},\;L_{1}=\frac{1}{HW}{\left\|x_{\mathrm{adv},i}-x_{i}\right\|}_{1}$ is the average $l_{1}$ distortion, and E [⋅] averages over samples i (and noises $z_{i}$), total generator loss [15]:

(19) $$L_{G}=L_{\mathrm{adv}}-\gamma E\left[D\left(f\left(x_{\mathrm{adv},i}\right)\right)\right]+\lambda_{\mathrm{TV}}L_{\mathrm{TV}}+\lambda_{1}L_{1},\;$$

where $p_{i}=\mathrm{softmax}\left(C\left(x_{i}\right)\right)$ and $q_{i}=\mathrm{softmax}\left(C\left(x_{\mathrm{adv},i}\right)\right)$ are the prediction vectors for sample i on clean and adversarial inputs, respectively, and KL(⋅‖⋅) is the Kullback–Leibler divergence.

## 3. Results

In this section, the performance of the proposed framework is empirically evaluated. We describe the data-acquisition process, hardware/software specifications, and comparative analyses conducted on both proprietary and public datasets to validate the system’s robustness.

### 3.1. Datasets

This study created and utilized an image dataset collected from real-world scenarios and conducted experimental validation by referencing publicly available datasets gathered across diverse scenarios. To ensure the reliability of the evaluation, we adopted a rigorous stratified sampling method [35] to divide the dataset as shown in Table 1. First, all image data were converted to grayscale and resized to 28 × 28 pixels. Then, to stabilize the data distribution, each image was independently normalized according to its mean and standard deviation. Specifically, 80% of the total data for each category was allocated as the training set, 10% as the test set for final performance evaluation, and another 10% as the validation set for hyperparameter tuning. The remaining images constituted an unlabeled training set to support the self-training process [36,37]. To ensure the rigor of the experimental conditions, we quantified the physical properties of the dataset. The digital tubes on the JK-50A unit utilize red LEDs with a peak wavelength ($\lambda_{p}$) of approximately 645 nm and a surface luminance of $150\pm 30\;\mathrm{cd}/m^{2}$. The environment is divided into 500–3000 lux.

### 3.2. Implementation Details

The whole training process in this work can be roughly divided into three stages. In the first stage, we build a small labeled dataset by randomly picking a few samples from each class, and train the Convolutional Neural Network (CNN)-SE classifier only on this set with cross-entropy and the Adam optimiser (learning rate 0.001, weight decay 0.0001, batch size 64) for 50 epochs; hence, we obtain a stable starting model. In the second stage, we extract global features from all training images and fit a PCA and k-means clustering algorithm (K-Means) pipeline [38] on these 64-dimensional features. This pipeline is not used as a classifier, but only to select a small, roughly balanced group of unlabeled candidates from each cluster in each self-training round. For each class, we sample K-labelled seeds and use K=2 in all main experiments, and we only accept pseudo-labels whose maximum softmax probability exceeds the confidence threshold [39] *τ* = 0.7. During self-training, candidates that stay near the center of their cluster and are predicted with high confidence are kept, their softmax outputs are treated as soft pseudo labels, and they are added to the dataset when we update the network for a few more epochs. From a later round, an Adversarial Generative Adversarial Network (AdvGAN++) module is turned on to slightly perturb the shared features and generate bounded adversarial examples, and the classifier head is then fine-tuned on mini-batches that mix clean and perturbed images before we finally fix the model. The training environment is shown in Table 2.

### 3.3. Comparative Experiment

To assess the effectiveness of the integrated model, several performance metrics—including accuracy, precision, and F1-score—are utilized. The following subsections present the recognition results, highlighting the model’s capability to handle multi-source digits under varying illumination.

#### 3.3.1. The Analysis of Accuracy

**Figure 4:**
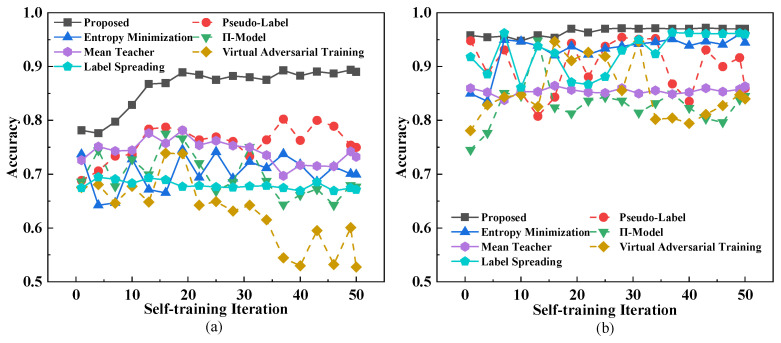
A contrastive analysis of model accuracy. (**a**) Diagram of Left: the dataset we collected in a background with 500~3000 lx. (**b**) Diagram of Right: Public dataset consisting of binary images, serving as an ideal baseline for moderate lighting (300~800 lx).

This study compares our proposed improved algorithm with the traditional self-training algorithm. The proposed model is evaluated against the traditional self-training model, respectively, employing both our collected dataset and the public display dataset for verification and performance comparison. As depicted in Figure 4.

The experiment result in Table 3 shows that the proposed model achieves higher accuracy than baseline methods in all evaluated datasets, including the public benchmark of digit display and the proprietary local collection.

#### 3.3.2. The Analysis of Robustness

**Figure 5:**
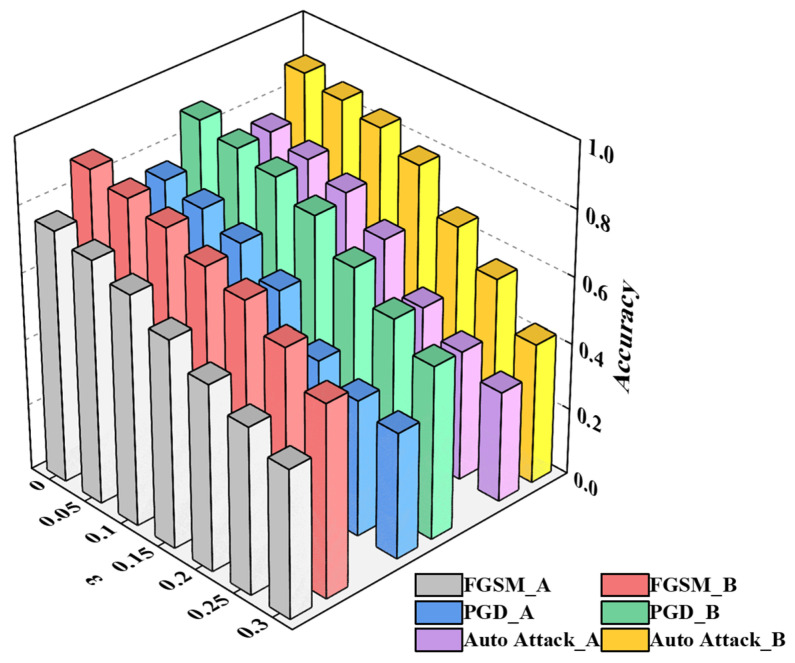
Model accuracy under different and varying degrees of attack. A: the model before adversarial training; B: the model after adversarial training.

To evaluate the model’s stability and generalization capability under noisy inputs and adversarial attacks, this study employs three adversarial attack methods based on the model after adversarial training for verification concerning FGSM, PGD, and Auto-Attack. Under varying attack intensity, the experiments demonstrate the variation in accuracy on the initial test set before and after adversarial training in Table 4, as well as the success rates of different adversarial attacks in Table 5, as shown in Figure 5.

From Table 4 and Table 5, it is evident that the model after adversarial training with AdvGAN++ demonstrates stronger anti-interference ability and higher accuracy compared to the original model. Demonstrate that the model after adversarial training with AdvGAN++ exhibits stronger anti-interference ability and higher accuracy compared to the original model.

In addition, the results demonstrate that the success rates of various adversarial attack algorithms increase with the increase in ε under identical model conditions.

### 3.4. Ablation Study

Ablation studies are conducted to investigate the contribution of each individual module within the framework. Specifically, we analyze the impact of the SE-Attention mechanism and the evolution of model performance across successive self-training iterations.

#### 3.4.1. The Ablation Analysis of Components

**Figure 6:**
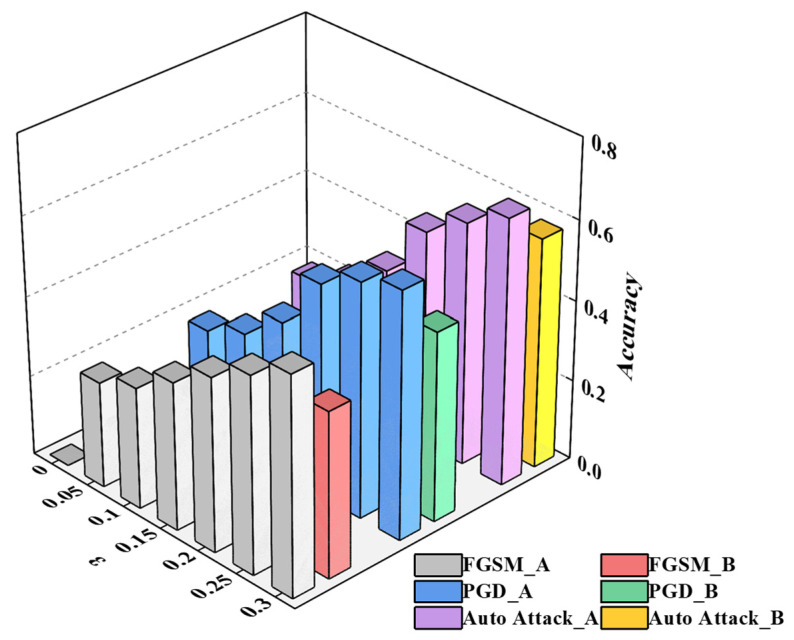
Model accuracy under different and varying degrees of attack. A: the model before adversarial training; B: the model after adversarial training.

To see how AdvGAN++ changes where the model looks, we compare SE attention heatmaps from a model without AdvGAN++ and a model with AdvGAN++ using a different view. In Figure 6, row 4, blue means the Not-AdvGAN++ model pays more attention there, and red means the AdvGAN++ model does.

**Figure 7:**
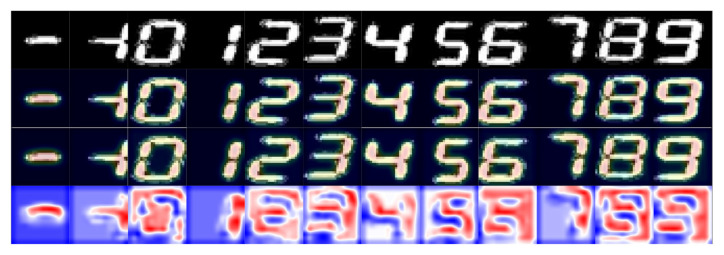
Attention heatmaps with and without adversarial training. Row 1: original seven-segment image; Row 2: attention without adversarial training; Row 3: attention with adversarial training; Row 4: difference map, blue indicates stronger attention in the non-adversarial, red indicates stronger attention in the adversarial model.

Visually, the model with AdvGAN++ demonstrates a stronger focus on the skeleton of the intended active seven-segment display [40], and on bends and junctions, yielding more continuous heat that better follows the strokes, while the model without AdvGAN++ tends to pay more attention to outer contours, unlit segments, and background. To evaluate the individual contributions of each module, we conducted an ablation study by removing components from the model and assessing the resulting accuracy, as shown in Figure 7.

Experimental results demonstrate that the attention mechanism significantly enhances recognition accuracy. In contrast, uniform sampling via the K-means algorithm yields limited effectiveness. Whilst this approach introduces a uniform sampling mechanism, it also presents considerable recognition challenges. This is because after clustering divides samples into distinct clusters, there is no guarantee that each cluster will yield the required samples. Should two different clusters produce identical samples, this effectively identifies the two most similar yet most dissimilar samples within the clustering dimension, thereby exacerbating the model’s recognition difficulty. However, introducing adversarial training mitigates the negative effects of equal-weight sampling, enabling the K-means algorithm to achieve high-precision recognition when combined with this method. Furthermore, the AdvGAN++ method effectively generates high-quality digital tube samples that meet the requirements, thereby enhancing the predictive capability of the recognition model. The results of the ablation study are shown in Table 6.

#### 3.4.2. Ablation Analysis on the Upper Limit for Pixel Changes

**Figure 8:**
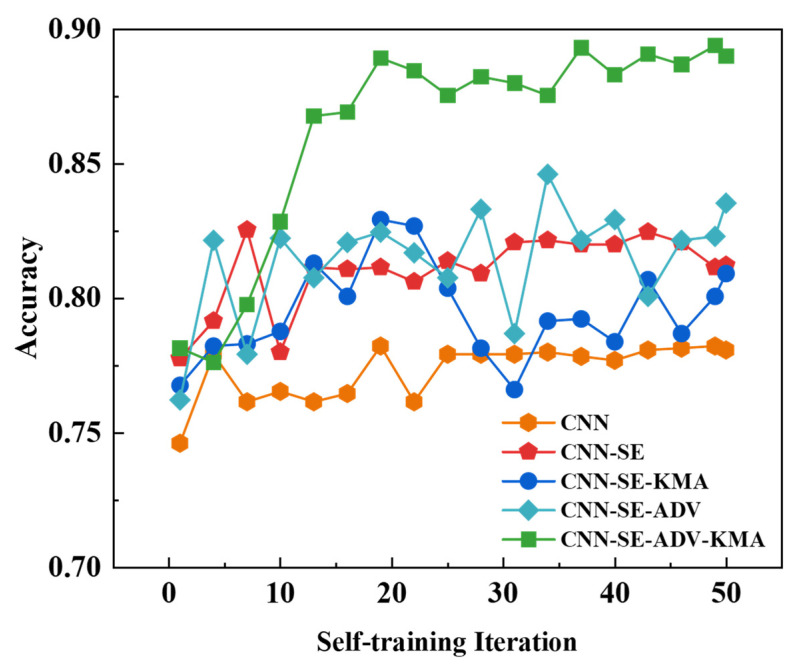
Ablation Map.

We investigated the impact of different pixel changes in the AdvGAN++ module on model accuracy before and after adversarial training, as shown in Figure 8.

**Figure 9:**
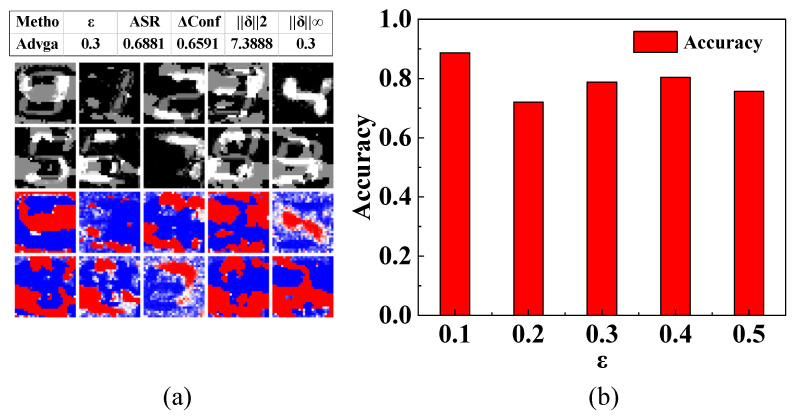
The ablation map of the *ε*. (**a**) Visualization of adversarial samples generated at ϵ=0.3. The first and second rows display the absolute pixel difference (perturbation) in grayscale; the third and fourth rows present the perturbation heatmaps, where blue indicates regions with minimal pixel modification and red indicates regions with significant perturbation intensity. (**b**) The impact of varying the pixel perturbation budget ϵ on the model’s recognition accuracy. ASR denoted as Attack Success Rate, ΔConf denoted as Confidence Erosion, ‖δ‖2 denoted as Mean L2 norm, ‖δ‖∞ denoted as Mean L∞ norm, *ε* denoted as per-pixel perturbation budget.

On the test set, we evaluated generative adversarial attacks under a 0.3-pixel budget in Figure 9a, with each image randomly initialized eight times and the strongest attack result retained. This attack resulted in 68.8% of samples being misclassified, reducing the model’s confidence in the true class by an average of 0.659. The average perturbation magnitude was 7.389, with a maximum single-pixel change of approximately 0.300, precisely reaching the upper limit of the budget. Relaxing the constraints may enhance attack strength. Under the same budget, this method significantly degrades classifier performance and induces high misclassification rates.

Experimental results indicate the model achieves peak accuracy at *ε* = 0.1, as shown in Figure 9b. We attribute this to the regularization effect induced by adversarial perturbations: moderate perturbations (*ε* = 0.1) function as data augmentation during testing, prompting the model to learn more robust feature representations through controlled noise introduction, thereby enhancing generalization capability. This level of perturbation introduces necessary diversity while preserving sufficient semantic information, enabling the model to capture more discriminative features. In contrast, perturbations with too small a level may fail to provide adequate regularization stimulus, whilst excessively large perturbations risk undermining the fundamental semantic structure of the image.

**Figure 10:**
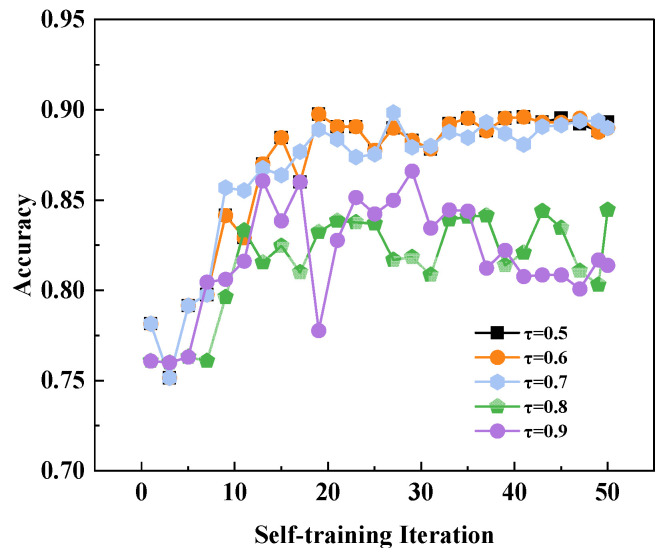
The ablation map of the *τ*. *τ* is denoted as the confidence threshold for pseudo-label selection.

To conduct further sensitivity analysis on the model, we performed an ablation experiment on the parameter. As shown in Figure 10, the model achieves high accuracy at (*τ* = 0.5, 0.6, 0.7), whereas accuracy declines at (*τ* = 0.8, 0.9). This stems from placing excessive trust in the model’s initial judgments. During the initial phase, the model’s performance remains immature and unstable. Granting it excessive self-decision weighting causes cognitive biases to amplify and accumulate progressively, ultimately preventing optimal performance. Therefore, assigning a moderate self-decision weight is crucial; in the initial stages, such weighting often yields significant performance improvements.

**Figure 11:**
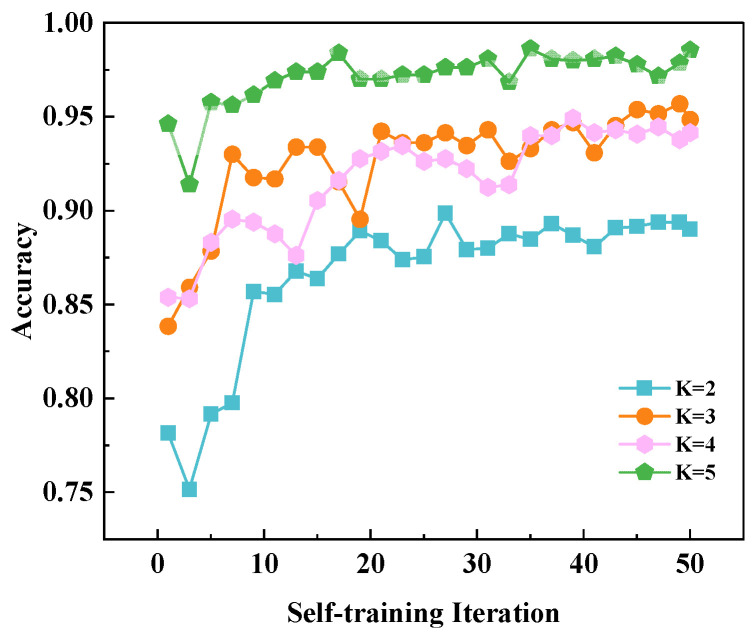
The ablation map of the *K*. *K* is denoted as the number of seed labels per class.

Experiments demonstrate that accuracy improves with increasing initial label count, and the model’s initial accuracy is positively correlated with the number of initial labels. In the initial stage of self-training, the model often acquires a number of erroneous samples, leading to a decline in accuracy. However, as adversarial training continues, the model’s ability to recognize correct samples and resist incorrect ones gradually strengthens, gradually easing the negative impact of the accumulation of original incorrect samples. The initial accuracy is positively correlated with the initial number of labels, but this does not imply that a model with fewer initial labels will have lower final accuracy than one with more. That is why, in the early days of self-training, the model’s ability to correctly label samples is super important; a good start can significantly boost the overall performance of the model. As illustrated in Figure 11, the (*K* = 3) curve achieves comparable or even superior final accuracy to the (*K* = 4) curve despite having fewer initial labels. This outcome largely stems from the acquisition of high-quality samples during the initial phase, which substantially improved the model’s subsequent performance.

## 4. Conclusions

This study proposes an improved semi-supervised learning framework that strategically combines several components to create a robust and efficient recognition system and, based on a Convolutional Neural Network enhanced with an SE-block mechanism, extracts discriminative features. The learning process is guided by a novel uniform sampling strategy applied to clustered unlabeled data, ensuring balanced training. To fortify the model against perturbations, we incorporate adversarial training by AdvGAN++. By combining a CNN-SE feature extractor with a K-means stratified sampling strategy and AdvGAN++ adversarial training, we established an automated workflow that handles data acquisition and model training with minimal human intervention. Our experiments show that this approach is highly effective under different lighting conditions, achieving 89.3% accuracy on the proprietary local dataset and 98.1% on public datasets. The practical value of this work lies in providing a non-intrusive “optical data diode” for devices like the JK-50A power supply that lack digital ports. This system maintains a strict physical air gap for cybersecurity while reducing manual labeling time and hardware retrofitting costs by over 90%.

To mitigate flicker, this study synchronized the camera’s exposure time to span multiple LED refresh cycles and utilized AdvGAN++ to model temporal banding artifacts, ensuring stable intensity even during rapid state transitions. To capture full values in high-speed displays without motion blur, our approach balances exposure duration with the display’s update rate. However, challenges remain for ultra-high-frequency PWM and non-standard safety protocols involving varying flicker frequencies. Future research will explore intelligent adaptive shutter synchronization and deep-learning-based de-banding algorithms to ensure robust recognition in complex, variable-frequency signaling environments.

## Figures and Tables

**Table 1:** Data partitioning.

Class	Local Dataset				Public Dataset			
	Train	Test	Val	Total	Train	Test	Val	Total
1	800	100	100	1000	2838	354	354	3548
2	800	100	100	1000	1598	201	199	1998
3	800	100	100	1000	1404	176	175	1755
4	800	100	100	1000	1928	242	241	2411
5	800	100	100	1000	1396	176	174	1746
6	800	100	100	1000	1647	207	205	2059
7	800	100	100	1000	1550	195	193	1938
8	800	100	100	1000	1448	181	181	1810
9	800	100	100	1000	1308	165	163	1636
−1	800	100	100	1000	-	-	-	-
-	800	100	100	1000	-	-	-	-
.	800	100	100	1000	-	-	-	-

In the local dataset, since −1 occupies one character on the LED display, −1 and - are treated as separate categories.

**Table 2:** Environment Configuration.

Item	Configuration
Operation System	Windows 11 Home
CPU	Intel Core i7-12700H (14 cores, 20 threads)
GPU	NVIDIA GeForce RTX 3050 Laptop GPU (4 GB)
Python	3.9.23
PyTorch	2.5.1
ML Libraries	scikit-learn 1.3.0/NumPy 1.24.3
Reproducibility	Random Seed = 42
Cuda sensor pixels	12.1 1080p

Driver runtime is CUDA 12.9 (NVIDIA driver 576.02); PyTorch uses cu121.

**Table 3:** Comparative analysis of improved models and base models.

Method	Local Dataset		Public Dataset	
	Test_Acc%	ΔLocal%	Test_Acc%	ΔPublic%
Proposed(ours)	89.3 ± 0.4	13.1	98.1 ± 0.2	1.3
Pseudo-Label	76.2 ± 0.8	0	87.4 ± 0.6	−9.4
Entropy Minimization	71.1 ± 1.1	−5.1	96.2 ± 0.5	−0.6
Π-Model	68.5 ± 0.9	−7.7	85.3 ± 0.7	−11.5
Mean Teacher	73.1 ± 0.7	−3.1	87.0 ± 0.5	−9.8
Virtual Adversarial Training	52.4 ± 1.5	−23.8	84.8 ± 0.8	−12
Label Spreading	67.3 ± 0.9	−8.9	96.8 ± 0.3	0

The best baseline on each dataset is used as a reference when computing Δ, ΔLocal, and ΔPublic, which denote the improvement over the best baseline on each dataset.

**Table 4:** Model Accuracy under different *ε* attacks.

*ε*	Clean			AdvGAN++		
	FGSM	PGD	Auto-Attack	FGSM	PGD	Auto-Attack
0	76%	76%	76%	89%	89%	89%
0.05	73%	73%	73%	86%	86%	86%
0.1	69%	69%	69%	84%	84%	84%
0.15	63%	61%	61%	79%	78%	78%
0.2	56%	47%	46%	75%	69%	66%
0.25	50%	41%	39%	68%	60%	56%
0.3	45%	38%	33%	58%	52%	42%

**Table 5:** Model Success Rate under different *ε* attacks.

*ε*	Clean			AdvGAN++		
	FGSM	PGD	Auto-Attack	FGSM	PGD	Auto-Attack
0	0	0	0	0	0	0
0.05	27%	27%	27%	14%	14%	14%
0.1	31%	31%	31%	16%	16%	16%
0.15	37%	39%	40%	21%	22%	22%
0.2	44%	53%	54%	25%	31%	34%
0.25	50%	59%	61%	32%	40%	44%
0.3	55%	62%	67%	42%	48%	57%

**Table 6:** The recognition performance of different algorithms on the test set.

Method	Test_Acc	Train_Loss
CNN	76%	0.0126
CNN-SE	80%	0.0136
CNN-SE-K-means	83%	0.0052
CNN-SE-K-means-AdvGAN++	89%	0.0029

The test set incorporates diverse samples.

## Data Availability

The datasets generated and analyzed during the current study are publicly available in the Zenodo repository at https://doi.org/10.5281/zenodo.17855380. The publicly available comparison dataset used in this work can be accessed at https://github.com/yhsc0001 (accessed on 18 October 2025).

## Abbreviations

The following abbreviations are used in this manuscript:

SE Block	Squeeze-and-Excitation B Block
AdvGAN++	Adversarial Generative Adversarial Network (Enhanced Version)
CNN	Convolutional Neural Network
K-means	K-means Clustering Algorithm
ReLU	Rectified Linear Unit
